# Transcranial direct current stimulation (tDCS) improves emotion regulation in children with attention-deficit hyperactivity disorder (ADHD)

**DOI:** 10.1038/s41598-024-64886-9

**Published:** 2024-06-17

**Authors:** Reza Estaji, Mariam Hosseinzadeh, Fariba Arabgol, Vahid Nejati

**Affiliations:** 1 Department of Psychology, Shahid Beheshti University, Tehran, Iran; 2 Department of Psychiatry, Shahid Beheshti University of Medical Sciences, Tehran, Iran; 3 Department of Psychiatry, Imam Hossein Hospital, Shahid Beheshti University of Medical Sciences, Tehran, Iran; 4 Behavioral Sciences Research Center, Shahid Beheshti University of Medical Sciences, Tehran, Iran

**Keywords:** Transcranial direct current stimulation (tDCS), Emotion regulation, Children with attention-deficit hyperactivity disorder (ADHD), Neuronal development, Paediatric research

## Abstract

Children with attention deficit/hyperactivity disorder (ADHD) typically exhibit difficulties in emotion regulation. It has been shown that the dorsolateral prefrontal cortex (dlPFC) and ventromedial prefrontal cortex (vmPFC) are crucially involved in these deficient processes. In this study, we aimed to explore the impact of electrical stimulation over the left dlPFC and right vmPFC on emotion regulation in children with ADHD. Twenty-four children with ADHD completed the Emotional Go/No-Go and Emotional 1-Back tasks while undergoing transcranial direct current stimulation (tDCS) in three separate sessions, each with a different electrode placement: anodal dlPFC (F3)/cathodal vmPFC (Fp2), anodal vmPFC (Fp2)/cathodal dlPFC (F3), and sham stimulation. During both real tDCS conditions, the accuracy of pre-potent inhibitory control and working memory performance improved, but not speed. This study provides evidence that the left dlPFC and the right vmPFC are involved in emotion regulation in ADHD.

## Introduction

Attention deficit-hyperactivity disorder (ADHD) is associated with various cognitive processing deficits. These deficits include impairments in perceptual^1^, attentional^2^, temporal^3^, emotional^4^, executive^5^, social^6^, and motivational^7^ processing. Two fundamental theories—dysexecutive theory^5^ and motivational theory^8,9^—have been developed to describe these cognitive impairments.

According to dysexecutive theory, individuals with ADHD struggle with impaired inhibitory control as a core executive function (EF), which involves secondary EFs including working memory, planning, problem-solving, and emotional self-regulation^5^. Among these, impaired emotional self-regulation has been shown to be a core component of ADHD, which interrupts top-down processing of in-hand information. This deficit may manifest as emotional impulsivity, characterized by quick and uncontrolled emotional reactions, as well as emotional dysregulation, involving dysfunctional top-down efforts to moderate the primary emotional reaction^10,11^.

The dual pathway theory suggests that individuals with ADHD experience impaired executive and emotional/motivational processes. Consequently, an inhibitory deficit leads to executive dysfunction and reduced task engagement, while dysfunctional reward/emotional and bottom-up attentional processing result in hypervigilance toward salient information and a lack of controllability^9^.

In line with the aforementioned neuropsychological theories of ADHD, impaired executive function in these patients is broken down into two types: cold and hot EFs. The former refers to difficulties in selecting, sustaining, shifting, and inhibiting information to guide goal-directed behavior^12^, while the latter are typically evoked by motivating and emotionally meaningful contexts^13^. While this categorization can encompass various cognitive impairments in individuals with ADHD, distinguishing between these two types still poses a challenge.

Nejati^14^ developed a matrix of higher cognitive functions based on types of information and processing styles, which categorizes these functions into four classes: hot, cold, warm, and cool cognition. Hot cognition involves intuitive processing of emotional stimuli, such as emotion recognition and social cognition. Cold cognition, on the other hand, refers to analytic processing of non-emotional stimuli including cognitive flexibility, working memory, and inhibitory control. Warm cognition indicates intuitive processing of non-emotional stimuli, such as risky decision-making and delay discounting, while cool cognition refers to analytic processing of emotional stimuli, like emotion regulation and cognitive biases.

With this theoretical standpoint, patients with ADHD experience various cognitive deficits across multiple domains. They show significant deficits in cold cognitive domains such as inhibitory control, working memory, and cognitive flexibility, leading to impaired goal-directed behaviors^15–18^. Moreover, they struggle with hot cognition tasks like facial emotion recognition^4^, and warm domains of cognition, including motivation control and reward-related decision making^19–22^. Additionally, individuals with ADHD face challenges in cool cognition areas, such as emotional response inhibition^23–26^ and emotional working memory^27,28^, which are dependent on their ability to regulate emotions^29^.

At the neural level, the ventromedial prefrontal cortex (vmPFC) is associated with intuitive and emotional processing, while the dorsolateral prefrontal cortex (dlPFC) is more commonly associated with analytical or logical processing^30^. According to a meta-analysis of functional magnetic resonance imaging (fMRI) studies, activation of the lateral prefrontal cortex (PFC) including the dlPFC and ventrolateral prefrontal cortex (vlPFC), and dorsal anterior cingulate cortex (ACC) during analytical cognitive tasks has been shown to be supported by executive control network, in which the dlPFC plays a key role^31^. On the other hand, social and emotion/reward-related information processing seems to be correlated with activation of the medial PFC, particularly the vmPFC^32^. The vmPFC interacts with the lateral PFC and ACC during executive processing of emotional/motivational information^32,33^. This implies that cognitive processing of this type of information relies on both the regions involved in cognitive control and those involved in emotional/motivational processes. A notable example of this kind of processing is emotion regulation, which relies on both the dlPFC and vmPFC, as it is dependent on executive control and emotional processing simultaneously^34^.

Recent neuroimaging studies investigating ADHD have revealed a decrease in task-positive activation not only in the neural circuits associated with cognitive control and working memory, such as the dlPFC and inferior frontal cortex (IFC), but also in neural networks involved in emotional and intuitive cognitive functions, such as the vmPFC, orbitofrontal cortex (OFC), and ventral anterior cingulate cortex (ACC). Additionally, patients with ADHD have been shown to exhibit increased activation of the default mode network (DMN), which is associated with mind-wandering and self-referential processing^22^. Notably, the DMN is one of the brain networks crucial for emotional and motivational processes, with the vmPFC serving as its central hub. The DMN interacts with another network called the central executive network (CEN), involved in cognitive control, with the dlPFC as a key region^35^.

Beyond correlational neuroimaging studies, non-invasive brain stimulation (NIBS) offers the opportunity to study brain function by modulating the excitability of target brain regions^36^. Transcranial direct current stimulation (tDCS), as an NIBS technique, alters cortical excitability by applying a direct electrical current over the scalp to the underlying cortical areas^37^. Recently, it has been widely used in ADHD studies to improve cognitive functions. Recent studies in ADHD patients have indicated that anodal tDCS over the left dlPFC combined with cathodal tDCS over the right vmPFC improves cold EFs such as inhibitory control^38,39^ and working memory^38,40^. Conversely, the reversed electrode placement has been shown to not only enhance response inhibition as a cold EF^40,41^ but also warm cognitions, including reward processing^42^. It appears that suppressing the left dlPFC activation through cathodal stimulation leads to activation of the right dlPFC, which is a key region in inhibitory control^41^. Additionally, anodal tDCS over the right vmPFC in these studies could improve the reduced task-positive activation of this region, which plays a significant role in motivational cognitive processes. Specifically, the underlying mechanism of reward processing improvement in ADHD children after receiving simultaneous cathodal stimulation of the dlPFC and anodal stimulation of the vmPFC is that this stimulation protocol promotes inhibitory control as a cold EF, which has a regulatory role in emotion/reward-related information processing^42,43^. On the other hand, activating vmPFC may also lead to a more rational estimation of reward value, which primarily involves warm cognition^42^.

Given the background provided, executive and emotional/motivational domains of cognition are impaired in ADHD due to reduced task-positive activity in the dlPFC and the vmPFC. Consequently, we assume that impaired emotion regulation, a cool cognition in children with ADHD, is associated with the involvement of the left dlPFC and the right vmPFC. Therefore, our aim is to explore the potential role of upregulating the dlPFC while downregulating the vmPFC, and vice versa, using tDCS to improve the impaired emotion regulation in these children.

## Materials and methods

### Participants

In the present study, we applied G*power to calculate the required sample size^44^. Considering a power of 0.95, an alpha level of 0.05, and an effect size of 0.40, proposed for tDCS studies^45^, the needed sample size for this study design was 16. We recruited eight more participants to compensate for unpredictable dropouts. The study included 24 children who were diagnosed with ADHD by a professional child psychiatrist (researcher/author (M.H.)) using the Diagnostic and Statistical Manual of Mental Disorders 5th ed.^46^. The participants were between 6 to 12 years old, with a mean age of 9.09 ± 1.58. Of the participants, 3 had mild ADHD, 19 had moderate ADHD, and 2 had severe ADHD, according to the SNAP-IV scale. None of the participants had a history of traumatic brain injury, neurological disorders, or any other major psychiatric disorders, as confirmed by the psychiatric clinical interview. All participants had normal vision and were right-handed. Almost half of them were taking medication such as methylphenidate, fluoxetine, and clonidine, which they stopped taking 12 h before the experimental sessions. The demographic parameters of the participants are shown in Table 1. The study was conducted in accordance with the ethical standards of the Helsinki Declaration^47^ (revised in 2013) and was approved by the ethical committee of Shahid Beheshti University.

**Table 1:** Demographic characteristics and ADHD rating of participants.

Variables	M (SD)
Demographic characteristics	
Age (years)	9.16(1.57)
Education (years^a^)	3 (1.66)
Gender (male/female)	18/6
SNAP-IV	28.70 (7.92)
ERC	60.54 (4.90)
BRIEF	108.55 (27.57)

Abbreviation: M: mean, SD: standard deviation.

^a^The number of academic years a person completed in a formal program.

### Swanson, Nolan, and Pelham rating scale (SNAP-IV)

**This scale has been developed in accordance with diagnostic criteria of ADHD in the DSM-IV^48^. It comprises 18 items, nine for inattention, six for hyperactivity, and three for impulsivity. The items are rated on a four-point Likert style scale to reflect the severity of symptoms in the last month. The results of the rating scale were used to confirm the diagnosis and determine the effectiveness of intervention through correlating the outcomes of this scale with following computerized tasks. The SNAP-IV has been found to be valid and reliable for use in the Iranian population in a previous study, with a Cronbach's alpha of 0.81 for hyperactivity/impulsivity and 0.75 for inattention^49^.

### Emotion regulation checklist (ERC)

This checklist was developed by^50^ as scale to measure emotion regulation in children aged 5 to 12. It consists of 24 items, with eight focusing on emotion regulation and 16 on lability/negativity, and is rated on a four-point Likert style scale. The results of this checklist were used to estimate participants' baseline emotion regulation status and the efficacy of the intervention. The ERC has been validated and shown to be reliable for applying in the Iranian population, Cronbach’s alpha 0.76 for lability/negativity and 0.69 for emotion regulation^51^.

### Behavior rating inventory of executive function (BRIEF)

This inventory measures different domains of executive functions relevant to real life situations including inhibition, shifting attention, emotional control, initiate, working memory, planning/organizing, organization of materials, and monitoring. It has 86 items rated on a three-point scale^52^. The results of this inventory were applied to determine participants’ baseline performance in executive functions and the effectiveness of stimulation protocols. In Iran, the BRIEF has been validated and found to be reliable with a Cronbach’s alpha of 0.93^53^.

### Emotional Go/No-Go task

The Go/No-Go task is applied to evaluate pre-potent inhibition^54^. During the task, participants are required to respond to the Go-stimuli but to withhold their response if a stop signal is presented immediately after the Go stimulus. In this study, the Go signals were presented within a frame, which appeared in one of 4 directions on the screen (right, left, up, and down) in each trial, and participants were asked to press the corresponding arrow key as quickly and accurately as possible. Each trial began with a 2000 ms fixation cross, followed by the appearance of the frame for 2000 ms. The interval between trials was 1000 ms. In a few of trials, an emotional picture emerged in the frame as No-Go signal, and participants had to refuse to response in those cases. Thirty percent of trails were No-Go ones, including an equal number of happy, sad and neutral faces. In this task, the accuracy and reaction time of Go and the accuracy of No-Go trials are the outcome measures. The main outcome measure of this test is the accuracy of No-Go trials, which was calculated separately for happy, sad, and neutral faces. We selected the pictures from the Nimstim set of facial expressions^55^ and standardized them for size (326 × 329 pixels). A laptop with a 15.6″ screen was used to present stimuli of all tasks at a viewing distance of about 50 cm. The task took approximately 6 min to complete.

### Emotional 1-back task

The emotional 1-Back test was developed to measure emotion-related working memory performance^56^. In this task, a sequence of stimuli is presented on the monitor and participants have to determine whether each stimulus is identical or non-identical to the previous one. In our study, there were 100 facial images as stimuli presented randomly with 30 subsequent identical stimuli as response. The target response images were happy, sad, and neutral faces, with 10 stimuli for each emotion. Each stimulus remained on the screen until response. Accuracy and reaction time were the outcome measures of this task as the index of working memory performance relevant to emotionally positive, negative, and neutral stimuli. The other details of stimuli including source, size, and presentation, were similar to those of the previous task.

### tDCS protocol

In the present study, we applied transcranial direct current stimulation by a saline-soaked pair of sponge rubber electrodes (25 cm^2^) of a battery-driven stimulator (ActivaTek Inc., USA). TDCS was conducted in three sessions, with electrodes arranged according to the 10–20 EEG international system, including: (1) anodal dlPFC (F3)/cathodal vmPFC (Fp2), (2) anodal vmPFC (Fp2)/ cathodal dlPFC (F3), and (3) sham stimulation. We applied a direct current of 2 mA for 20 min with 30 s ramp up and down in real conditions. For sham stimulation, the electrode position was the same as which used for real stimulation; however, the current was ramped down after 30 s without participants’ awareness. This procedure is commonly followed in tDCS studies, does not exert prolonged effects on cortical excitability, and is appropriate for blinding purposes^57,58^.

### Procedure

**Figure 1:**
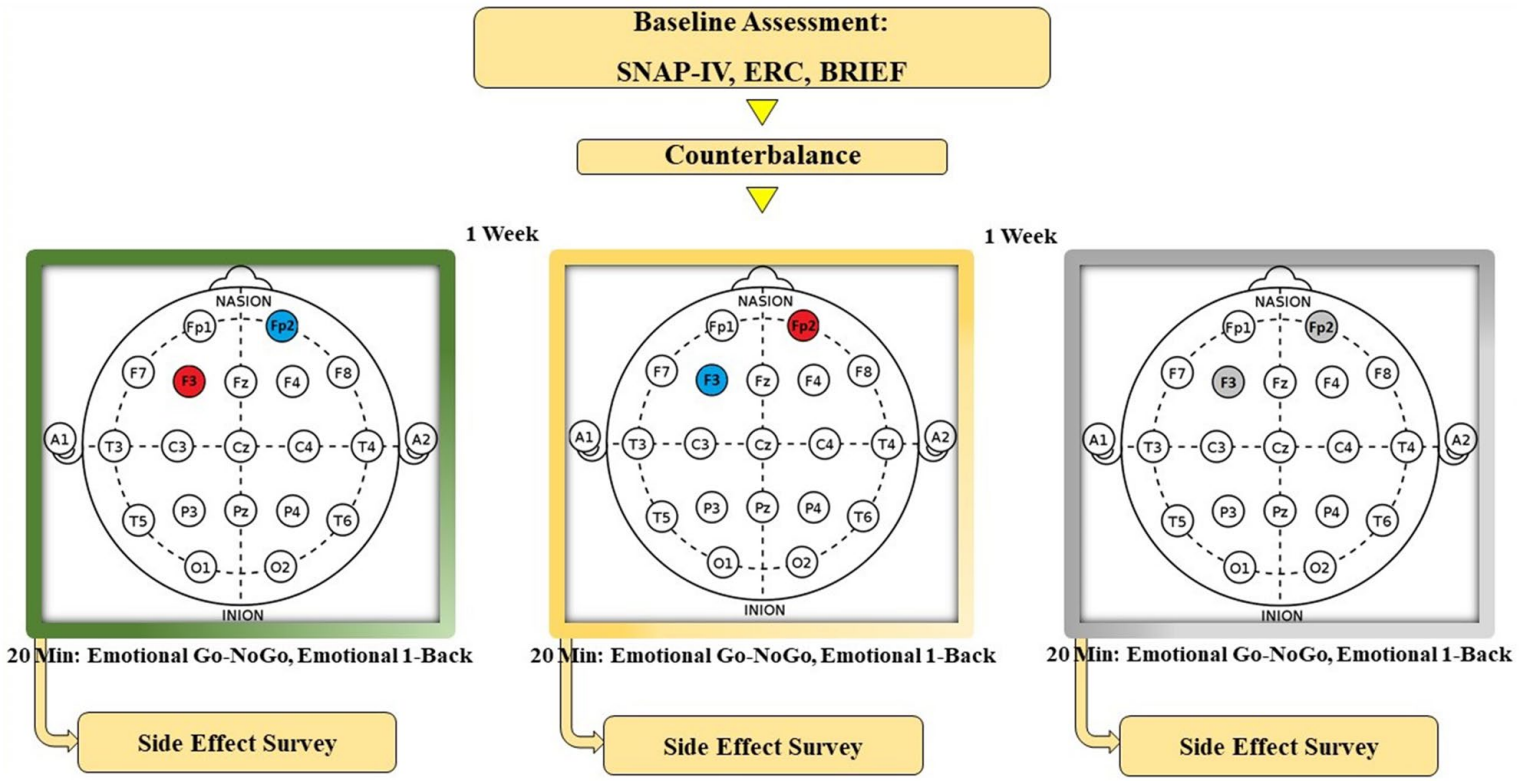
Experimental procedure. In the present study, participants received one of the tDCS protocols in a randomized order. Emotional Go-NoGo and emotional 1-Back tasks were performed in each session 5 min after stimulation. Finally, side effect questionnaire was completed.

**Figure 2:**
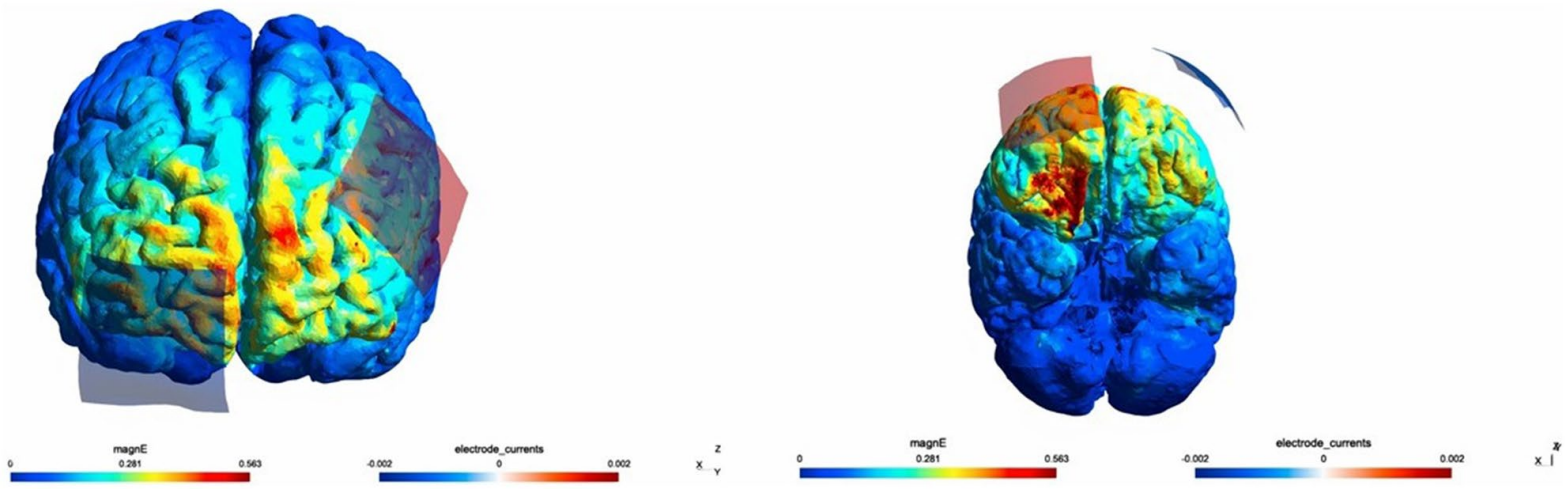
Distribution of electrical field was computed using SimNIBS. In this study, two 5 * 5 cm electrodes were positioned over F3 and Fp2, and the current intensity was 2 mA.

A single-blinded and complete crossover design was followed in this study. Moreover, a sham-controlled within-subject design was conducted in which all participants served as their own control, a design that increases statistical power substantially. To prevent the carry-over effects of previous stimulations, there was a one-week inter-session interval. We counterbalanced the order of the three stimulation conditions across participants. The stimulation duration was 20 min. Participants performed the Emotional Go/No-Go and Emotional 1-back tasks five minutes after the beginning of stimulation, which lasted for about 15 min. They were instructed to complete the tasks as accurately and quickly as possible. One researcher/author (R.E.) performed both, tDCS and computerized tasks. After each session, the participants completed a side-effect checklist^59^ and guessed what type of stimulation they received (real or sham). The participants were blinded to the stimulation condition, and their parents signed an informed consent form, Figs. 1 and 2.

### Data analysis

The statistical package SPSS for Windows version 23 was applied for data analysis. The normality of the data collected in each stimulation condition was confirmed using the Kolmogorov–Smirnov test. To examine the effect of tDCS on task performance, we conducted a one-way analysis of variance (ANOVA) with repeated measures for the within-subject factor of "Stimulation Condition". The dependent variables were the No-Go accuracy in the Emotional Go/No-Go task and the accuracy and reaction time in the Emotional 1-back task. For the unemotional outcome parameters, such as the Go accuracy and Go reaction time in the Emotional Go/No-Go task, we performed a one-way ANOVA with repeated measures for the within-subject factor of "Stimulation Condition" (3 different montages).

To test for data sphericity, Mauchly’s Test of Sphericity was administered and degrees of freedom were corrected using the Greenhouse–Geisser method, if required. In case of significant results in the respective ANOVAs, Fisher's LSD test was used as the post hoc analysis. Additionally, we included session and task order as well as medication use as covariates in an extra ANCOVA. A p-value of < 0.05 was considered statistically significant.

## Results

All participants tolerated the stimulation with no major side effects. During the first seconds of stimulation some slight and tolerable sensation of itching, tingling and burning under the electrodes were reported by the participants. Table 2 indicates mean and standard deviation (SD) of side effects in different tDCS conditions. Based on one-way ANOVAs, there were no significant differences between conditions for any of the respective side effects. Regarding to blinding, the guesses about the experimental conditions were lower than chance (44% correct, χ2(1) = 1.37, p = 0.24).

**Table 2:** Side effects of tDCS (means and SD) in the different stimulation conditions and the results of the respective ANOVAs.

Measures	Conditions, M(SD)			Statistics			
	dl/vm	vm/dl	Sham	df	F	P	ηp2
Pain	.21 (.65)	.28 (.08)	.00 (.00)	1.22	1.49	.23	.06
Vertigo	.00 (.00)	.00 (.00)	.00 (.00)	2	.	.	.
Burning	1.04 (.99)	1.46 (1.21)	.79 (1.28)	2	2.29	.11	.09
Tingling	.58 (.77)	1.04 (1.16)	.88 (1.22)	2	1.67	.19	.06
Confusion	.00 (.00)	.00 (.00)	.00 (.00)	2	.	.	.
Drowsiness	.65 (.21)	.63 (.17)	.63 (.17)	2	.03	.96	.002

Abbreviations: M: mean, SD: standard deviation, dl: dorsolateral prefrontal cortex, vm: ventromedial prefrontal cortex, areas pre and post the dash indicate anodal and cathodal electrode placement, respectively. df: degrees of freedom, F; F-value, P: P-value, ηp2: partial eta squared.

For the Emotional Go/No-Go task Table 3 provides data on the descriptive statistics of accuracy and reaction time for Go trials, and respective outcomes for No-go accuracy are shown in Table 4. Repeated measures ANOVAs were conducted to test the stimulation effects on task performance. Regarding No-Go accuracy, the ANOVA demonstrated a significant main effect of stimulation conditions (F_1.4_ = 5.93, p = 0.01, ηp^2^ = 0.20). Based on LSD post hoc, the results of anodal dlPFC/cathodal vmPFC stimulation and the reversed electrode placement differed significantly from the sham stimulation (MD = 6.67, p = 0.03), and (MD = 8.19, p = 0.01), respectively. Thus, under both real stimulation protocols compared to sham condition pre-potent inhibition increased. Moreover, one-factor ANOVAs performed for accuracy and reaction time of the Go stage indicated not significant main effect for accuracy (F_2_ = 0.72, p = 0.48, ηp^2^ = 0.03), and reaction time (F_2_ = 1.38, p = 0.26, ηp^2^ = 0.05).

**Table 3:** ANOVA results of the outcome measures in different stimulation conditions.

Measures	Total Score, M(Sd)			ANOVA Results			
	dl/vm	vm/dl	Sham	df	F	P	ηp2
Go accuracy	83.39 (15.31)	83.69 (12.48)	81.79 (12.61)	2	.72	.48	.03
Go reaction time	1.31 (.10)	1.33 (.11)	1.28 (.17)	2	1.38	.26	.05

Abbreviations: M: mean, SD: standard deviation, dl: dorsolateral prefrontal cortex, vm: ventromedial prefrontal cortex, areas pre and post the dash indicate anodal and cathodal electrode placement, respectively. df: degrees of freedom, F; F-value, P: P-value, ηp2: partial eta squared.

**Table 4:** The result of two factorial ANOVA on the study measures.

Measures	Total Score, M(Sd)	ANOVA Results					
	dl/vm	vm/dl	Sham	df	F	P	ηp2
No-go Accuracy	90.71 (7.12)	92.21 (8.41)	84.03 (16.68)	1.4	5.93	.01	.20
1-back Accuracy	86.11 (9.46)	89.26 (9.39)	79.03 (15.77)	1.54	8.23	.002	.26
1-back Reaction Time	1.15 (.19)	1.10 (.20)	1.15 (.19)	2	1.28	.28	.05

Abbreviations: M: mean, SD: standard deviation, dl: dorsolateral prefrontal cortex, vm: ventromedial prefrontal cortex, areas pre and post the dash indicate anodal and cathodal electrode placement respectively. df: degrees of freedom, F; F-value, P: P-value, ηp2: partial eta squared.

**Figure 3:**
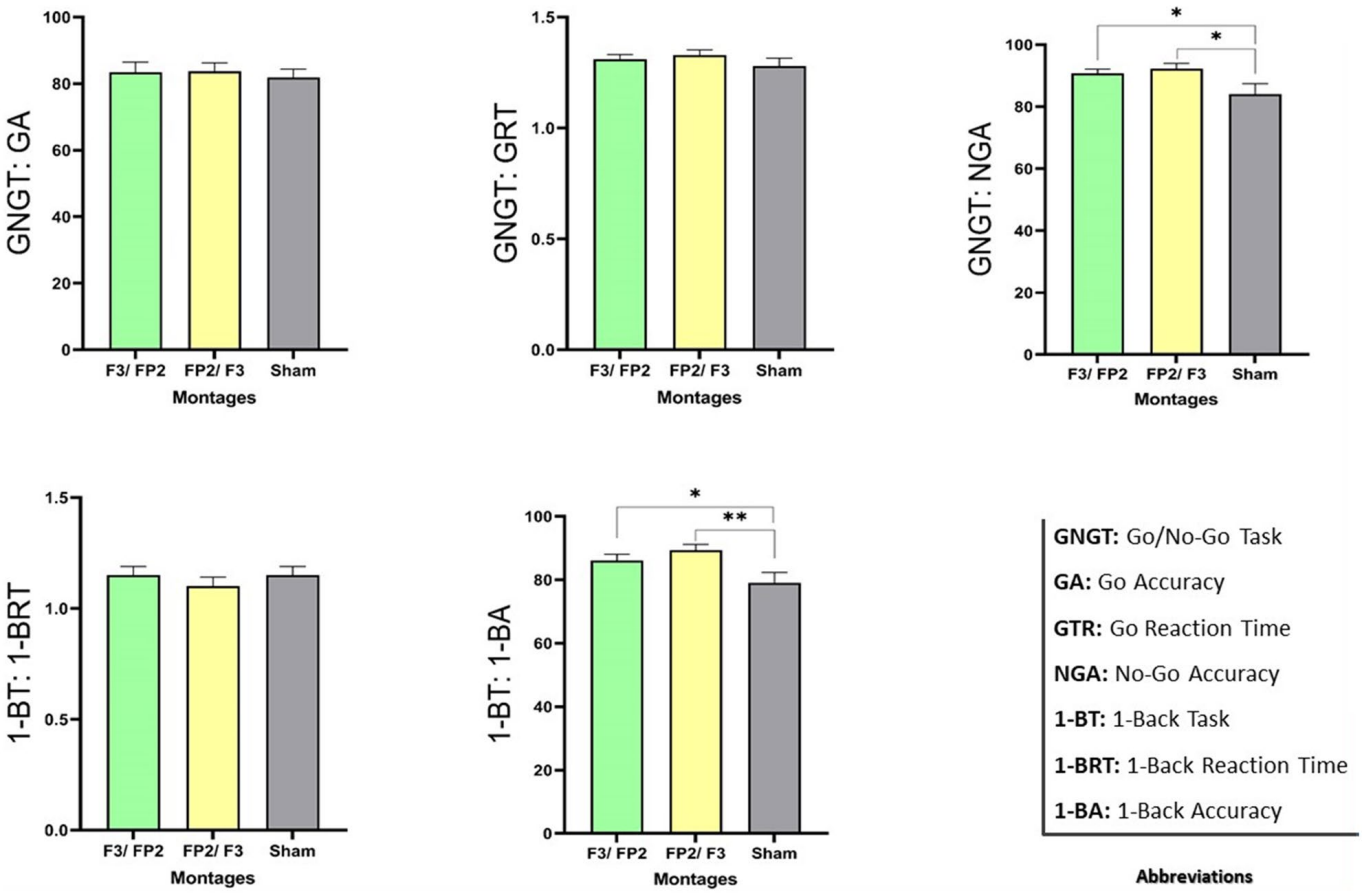
Effects of tDCS on emotional pre-potent inhibition and emotional working memory. * and **: significant at the level of 0.05 and 0.01 respectively based on the results of the pairwise comparisons between stimulation conditions.

The ANOVA performed for the Emotional 1-Back test accuracy indicated a significant main effect of stimulation (F_1.54_ = 8.23, p = 0.002, ηp^2^ = 0.26). The LSD post hoc results showed larger accuracy during both anodal dlPFC/cathodal vmPFC (MD = 7.083, p = 0.029), and anodal vmPFC/cathodal dlPFC stimulation (MD = 10.278, p = 0.001) compared to the sham condition. However, for the reaction time of the Emotional 1-Back task, the ANOVA results showed no significant main effect of stimulation (F_2_ = 1.28, p = 0.28, ηp^2^ = 0.05), Fig. 3.

No significant correlation was observed between the scores of the rating scales and emotional tasks. Finally, there were no significant effects for the co-variates session order, task order, and drug use for any of the dependent variables.

## Discussion

The results of the present study indicate significant improvement of emotional pre-potent inhibition and emotional working memory in ADHD children during both anodal tDCS over the left dlPFC and cathodal tDCS over the right vmPFC, as well as with the reversed electrode arrangement. Specifically, both real stimulation setups improved accuracy in the No-Go and 1-back tasks, which were our main outcome measures. These results were independent of participants’ baseline performance on rating scales of ADHD severity, emotion regulation, and executive functions.

### Stimulation condition impact

The bipolar electrode placement used makes it challenging to ascertain the specific role of each targeted stimulation area. Consequently, the results of the study are discussed in comparison to similar brain stimulation studies conducted on individuals both with and without ADHD.

In this study, we observed improved performance in emotional pre-potent inhibition and working memory during anodal left dlPFC and cathodal right vmPFC stimulation. This aligns with previous studies’ findings where anodal tDCS over the left dlPFC paired with cathodal tDCS over the right vmPFC led to enhanced inhibitory control^38,39^ and working memory^38,40,60^. The improvement in task performance observed in our study may be attributed to increased excitability of the left dlPFC and suppression of hyperactivity in the right vmPFC. Recent neuroimaging studies have demonstrated the critical role of the left dlPFC in analytic cognitive functions. Patients with ADHD often exhibit attenuated activity in this region during cognitive control tasks^61,62^. Additionally, as mentioned earlier, individuals with ADHD often show diminished deactivation of vmPFC while performing cognitive tasks, which can disrupt the top-down processing of information^63^. A plausible explanation for the improvement observed in emotional pre-potent inhibitory control and working memory during anodal tDCS over the left dlPFC and cathodal tDCS over the right vmPFC in our study is that this protocol may enhance reduced task-positive activation of the dlPFC during top-down processing of emotional stimuli. Additionally, Cathodal tDCS over the right vmPFC could reduce hyperactivity in this region, decreasing the likelihood of emotional stimuli interrupting cognitive control. Notably, aside from the dlPFC and vmPFC, which were target regions of stimulation in our study, other cortical areas may have also been influenced by the stimulation. Therefore, applying anodal stimulation over the left dlPFC might increase excitability of the dorsal ACC, which plays a significant role in analytic cognitions. Similarly, using cathodal stimulation over the right vmPFC could reduce activation of the PCC, whose dysfunctional activity in ADHD patients might interfere with attention control^43,64^.

As previously mentioned, the emotional-executive tasks used in this study require both analytic and emotional processes. Therefore, the results of this study can also be explained based on emotional processing and its neurocognitive basis. Previous functional imaging studies have shown the vmPFC and the dlPFC interact with each other concerning emotional processing^65^. Specifically, the vmPFC is involved in attributing arousal to emotional stimuli^66^, while the dlPFC engages in evaluating the valence of that information^67^. Thus, emotional stimuli appear to modulate executive functions, capturing attentional resources and either facilitating or slowing down executive information processing. On the other hand, executive functions can have a regulatory impact on emotional processing and regulate emotional responses^60^. In ADHD patients, it has been reported that emotionalizing stimuli of neuropsychological tasks may attenuate their performance in attentional control^68^, response inhibition^24–26,69^, and working memory^27,28^. Our research indicates that applying anodal tDCS over the left dlPFC can reduce valance attribution, make emotional stimuli less salient, and facilitate cognitive control over emotional stimuli leading to more regulated responses during emotional versions of inhibitory control and working memory tasks^60^.

This results can also be discussed in the context of the dysexecutive and dual pathway theories of ADHD^5,9^. It is observed that, anodal stimulation of the left dlPFC, combined with cathodal stimulation of the right vmPFC, enhanced emotional inhibitory control and emotional working memory. Improving inhibition provides great cognitive control over emotional stimuli and helps prevent emotional impulsivity, which is the initial step in emotional regulation. Additionally, enhancing working memory capacity can lead to a better ability to retain task instructions and respond more effectively to emotional stimuli^5,42^. According to the dual pathway model, the underlying mechanism of task improvement following anodal dlPFC and cathodal vmPFC stimulation is that increasing left dlPFC excitability can enhance executive control over the exaggerated emotional cues. Moreover, modulating the reduced deactivation of the right vmPFC in the first protocol may reduce hyper-vigilance to emotional stimulus and facilitate their control.

The results of the present study also indicated that cathodal stimulation over the left dlPFC and anodal stimulation over the right vmPFC using tDCS enhances participants’ performance in emotional tasks involving pre-potent inhibition and working memory. This finding replicates the results of previous studies, such as those^40,41^ and^42^ where cathodal stimulation of the left dlPFC combined with anodal stimulation of the right vmPFC improved analytic and emotional/intuitive cognitions, respectively. Indeed, this protocol appears to elevate the reduced activity of the the right dlPFC, a core hub in inhibitory control, through transcallosal connections, leading to higher cognitive control—an essential requirement for emotional tasks in the present study^41,43,62^. Furthermore, anodal stimulation over the right vmPFC in this protocol may ameliorate the reduced task-positive activation of this region, enhancing the capability of ADHD patients to more precisely estimate the true value of emotional stimuli and complete emotional tasks with greater control^22,42^.

Concerning the emotional demands of respective tasks, anodal stimulation of the right vmPFC can alter the arousal rating of emotional stimuli, weaken their chance to capture attentional resources, promote executive control over them, and finally lead to more accurate response^60^.

Viewed through the lenses of the dysexecutive and dual pathway theories, enhancing inhibitory demands in our task via cathodal stimulation of the left dlPFC paired with anodal stimulation of the right vmPFC fosters greater cognitive control over emotional stimuli and prevents impulsive, emotional reactions. Moreover, improving the accurate estimation of the value and arousal of emotional stimuli by upregulating the right vmPFC and downregulating the left dlPFC facilitates participants' ability to engage in top-down and effortful moderation of their primary emotional reactions, which constitutes a crucial aspect of emotional regulation^5,42^. This stimulation protocol also ameliorates impaired bottom-up emotion/reward-related processes and reduces arousal of the emotional cues. Therefore, they are less likely to receive large amount of attentional resources^9^.

Apart from mentioned studies, there are some other studies with contrasting results. For example, in a recent tDCS study on children with ADHD, anodic stimulation of bilateral dlPFC did not result in improved performance in attention, working memory and inhibitory control. A possible explanation for this lack of tDCS effect is that bilateral anodal stimulation of dlPFC could lead to counteracting of these two regions due to the inhibitory links between the left and right dlPFC^70^. Another tDCS study with no positive effect on adolescents with ADHD showed that anodal stimulation over rIFC did not improve ADHD symptoms and cognitive functions. A serious limitation of that study is that experimental and control groups were not balanced and there could be found some significant difference between them regarding demographic features, ADHD severity and comorbid disorders. Moreover, current intensity in that study was not enough to modulate ADHD symptoms and cognitive domains mediated by rIFC^71,72^. The findings of a more recent study on children and adolescents with ADHD indicated that there was no positive impact of anodal left dlPFC/ cathodal right vmPFC stimulation on neuropsychologic measures. That last study and our study are similar in terms of stimulation sites and current intensity. However, there is contradiction between the results of the studies and there are some plausible reasons for that. In the study of Guimaraes et al.^73^, small sample size is a matter of debate. Furthermore, although participants age range is wide (6–15) including children and adolescents, some features of the stimulation such as current intensity, stimulation duration and electrodes size are the same for all of them and this is not clear how tDCS might have affected their neuropsychological performance. Therefore, it is difficult to compare the result of the present study with study of Guimaraes et al.^73^.

### Relevance of baseline status in rating scales

In this study, we employed rating scales to assess the participants' baseline performance regarding ADHD severity, executive function, and emotion regulation. Moreover, we calculated the correlation between baseline performance and the outcomes of the computerized tasks to assess the efficacy of tDCS based on baseline performance. The findings from previous studies investigating the impact of baseline status on the effectiveness of NIBS are inconclusive. For example, tDCS has been shown to influence inhibitory control in children with ADHD, partly depending on symptom severity^74^. Conversely, a transcranial magnetic stimulation (TMS) study on patients with Myalgia Encephalomyelitis revealed that TMS could mitigate Fatigue Symptom regardless of symptoms severity^75^. The results of the present study indicated that tDCS could improve emotion regulation in children with ADHD, independent of their baseline state in rating scales. These results may suggest that tDCS could improve cognitive dysfunctions in children with ADHD independent of their baseline performance. This study provides evidence for the neural correlates of emotion regulation and paves the way for future research to incorporate emotion regulation into the neuropsychological evaluation of children with ADHD. However, it should be noticed that the majority of our participants had moderate ADHD. Therefore, research is warranted to explore the impact of patients' baseline status on the effectiveness of NIBS.

### Limitation and future direction

The present study has several limitations that merit consideration. We applied combined montages for tDCS, targeting two regions that play a potential role in emotion regulation in ADHD patients. Consequently, drawing a definitive conclusion regarding the specific contribution of each area to task performance is not feasible. Future investigations should explore stimulation montages that are specific to each area. Moreover, the interaction type between the dlPFC and vmPFC in emotion regulation in individuals with ADHD is another issue that should be addressed. In this study we applied an online and single-session tDCS with neuropsychological tasks. Repetitive stimulation sessions with follow-up are proposed for future studies to explore the suitability of this intervention for clinical application. Lastly, there were some methodological limitations such as the single-blinded design and the relatively limited sample size. It is worth mentioning that the current study is an exploratory study with a small sample size, and we cannot conclude the effectiveness of tDCS for clinical interventions.

## Conclusion

Individuals with ADHD commonly exhibit an impaired emotion regulation, which is associated with reduced task-positive activation in both the dlPFC and vmPFC at the neural level. These areas are crucial for executive processing of emotional content. This study aimed to explore if activating the vmPFC while suppressing the dlPFC, and vice versa, using tDCS improves the emotion dysregulation. Emotion regulation improvement was found during both stimulation protocols. In sum, we found anodal left dlPFC/cathodal right vmPFC and the reversed electrode placement stimulation improves emotion regulation in children with ADHD. Targeting these regions in future therapeutic studies might be promising to innovate interventional protocols to modulate emotion regulation in patients with ADHD. Moving forward, our findings support the consideration of anodal left dlPFC/cathodal right vmPFC and vice versa stimulation in multi-session interventions aimed at enhancing executive functions and emotion regulation in individuals with ADHD in future studies.

## Data Availability

The datasets generated during and/or analyzed during the current study are available from the corresponding author on reasonable request.
